# How and When Employees’ Growth Mindset Promotes Proactive Behavior: Alleviating Workplace Anxiety Under Time Pressure

**DOI:** 10.3390/bs16061009

**Published:** 2026-06-16

**Authors:** Yi Chen, Remila Abudurexiti, Jing Zhao, Huan Yang

**Affiliations:** 1 School of Labor and Human Resources, Renmin University of China, Beijing 100872, China; chenyi2023@ruc.edu.cn; 2 School of Business Administration, Xinjiang University of Finance and Economics, Urumqi 830012, China; 3 School of Economics and Management, Shihezi University, Shihezi 832003, China; yangh379@mail2.sysu.edu.cn

**Keywords:** job demands–resources (JD–R) model, growth mindset, workplace anxiety, proactive behavior, time pressure

## Abstract

Background: In increasingly dynamic and uncertain organizational environments, employees’ proactive behavior—characterized by self-initiation, future orientation, and change orientation—is critical for organizational adaptability and long-term competitiveness. Prior research has primarily examined how externally provided job resources stimulate proactive behavior. More recent work has begun to consider employees’ personal resources, but it largely adopts a capability level-based view, conceptualizing them as self-evaluations of individuals’ ability to control and influence their environment. This focus overlooks capability malleability-based personal resources that shape more fundamental beliefs about the malleability of human capability. Objective: Drawing on the job demands–resources (JD–R) model, this study investigates how employees’ growth mindset—reflecting beliefs that human capability can be developed—promotes proactive behavior by alleviating workplace anxiety, an anticipatory emotional state rooted in concerns about future work-related threats. We further examine time pressure as a key boundary condition. Method: A three-wave, multisource survey design was employed, collecting data from 326 employee–supervisor dyads. Results: The results show that employees’ growth mindset is negatively associated with workplace anxiety, which in turn positively predicts proactive behavior. Moreover, time pressure strengthens both the anxiety-buffering effect of growth mindset and the indirect effect of growth mindset on proactive behavior via workplace anxiety. Conclusions: By incorporating capability malleability-based personal resources into the JD–R model, this study advances understanding of the antecedents of proactive behavior beyond capability level-based self-evaluations toward deeper beliefs about the malleability of human capability. Applications: This study offers practical implications for managers seeking to cultivate employee proactivity.

## 1. Introduction

In the context of rapid technological change and increasing environmental uncertainty, organizations face growing pressure to sustain competitiveness through continuous adaptation. Under such conditions, employees’ passive task execution is no longer sufficient to support organizational development. Instead, organizations increasingly rely on employees’ proactive behavior—such as anticipating changes, solving problems, and modifying work practices—to build and renew dynamic capabilities (Bindl & Parker, 2011). Despite this growing demand, contemporary workplaces have witnessed the rise of disengagement-related phenomena—often labeled as “lying flat” or “quiet disengagement”, characterized by reduced initiative and minimal effort, resulting in persistently low levels of employee proactivity. This trend stands in sharp contrast to organizations’ growing demand for highly proactive employees. Accordingly, from a human resource development perspective, understanding how to sustainably enhance employees’ proactive behavior in today’s demanding work environment remains a theoretically and practically important research question (Cai et al., 2019).

Proactive behavior is widely recognized as a resource-intensive activity. Unlike routine task execution, proactive actions such as initiating change, improving procedures, and addressing potential problems require employees to invest substantial cognitive, emotional, and behavioral resources (Urbach & Weigelt, 2019). Consequently, scholars have increasingly adopted a resource-based perspective to examine the antecedents of proactive behavior (M. Han et al., 2024; Ouyang et al., 2019; Zhang et al., 2023). Existing research has predominantly focused on externally provided job resources, investigating how organizational human resource management systems and leadership practices enable employee proactivity. For example, studies have shown that change-oriented HRM systems (H. W. Lee et al., 2019), high-performance HRM systems (Lin et al., 2022), and leadership styles such as transformational leadership (Schmitt et al., 2016), empowering leadership (Schilpzand et al., 2018), and inclusive leadership (Xu et al., 2021) can foster employee proactivity by providing supportive work environments and developmental opportunities.

However, employees’ proactive behavior is shaped not only by external job resources but also by individuals’ internal resources. Accordingly, recent research has increasingly examined personal resources as important antecedents of proactive behavior, including resilience (Caniëls & Baaten, 2019), awareness regulation capacity (Hu et al., 2025; Zhang et al., 2023, 2024), and self-efficacy (Abuelhassan & AlGassim, 2022; Gerçek & Özveren, 2025). These constructs are predominantly capability level-based, reflecting individuals’ current perceived ability to control and perform effectively in their environment. This capability level-based perspective, however, captures only one layer of internal psychological functioning. In contrast, relatively little attention has been paid to capability malleability-based personal resources that operate at a more fundamental level by shaping individuals’ assumptions about the nature of human capability itself.

Growth mindset—defined as the belief that abilities can be developed through sustained effort and learning (Dweck, 1999)—represents a prototypical capability malleability-based personal resource. Unlike capability level-based resources, which reflect what individuals perceive they currently possess, a growth mindset captures whether individuals view capability itself as fixed or malleable over time. This distinction is theoretically important because it suggests that employees’ responses to job demands may depend not only on their perceived current abilities, but also on how they fundamentally construe the future nature of human capability. Growth mindset has gained increasing attention in organizational contexts, particularly in human resource development (S. J. Han & Stieha, 2020). For example, since assuming leadership in 2014, Microsoft CEO Satya Nadella has actively cultivated a growth mindset-oriented organizational culture by emphasizing learning over knowing in recruitment, promotion, and performance expectations. Employees are encouraged to continuously share learning progress rather than merely demonstrate existing expertise. Research similarly indicates that a growth mindset reshapes employees’ beliefs about effort, attributional responses to setbacks, and attentional allocation strategies (Keating & Heslin, 2015), implying its potential to promote proactive behavior. Nevertheless, prior research has not directly examined the relationship between growth mindset and proactive behavior. This gap limits our understanding of how employees’ capability malleability-based personal resources may drive proactive action. To address this, we draw on the job demands–resources (JD–R) model to develop a theoretical framework linking employees’ growth mindset to proactive behavior.

Originally proposed by Demerouti et al. (2001), the JD–R model posits that job characteristics can be broadly categorized into job demands and job resources. Early applications of the model primarily examined how demands and resources influence burnout and work engagement, which subsequently affect employee behavior, performance, and well-being. More recent research has extended the JD–R model by incorporating personal resources alongside job resources, thereby broadening its explanatory scope for employee behavior (Bakker & Demerouti, 2017; Bakker et al., 2023; Bakker et al., 2014). Building on this extended perspective, we conceptualize growth mindset as a valuable personal resource that shapes how individuals interpret and respond to job demands (Caniëls et al., 2018). According to the JD–R model’s health impairment process, when personal resources are insufficient, employees struggle to cope with job demands, resulting in negative psychological experiences such as burnout (Bakker & Demerouti, 2017), typically manifested as emotional exhaustion, depersonalization, and reduced personal accomplishment (Demerouti et al., 2001). In contrast, adequate personal resources enhance employees’ capacity to manage job demands (Demerouti et al., 2001), which mitigates such negative psychological states. In this study, we focus on workplace anxiety, an anticipatory emotional state rooted in concerns about future work-related threats (McCarthy et al., 2016), as a key affective response under such conditions. This choice is consistent with our logic, as both growth mindset and anxiety involve future-oriented considerations. Importantly, anxiety does not necessarily result from severe psychological dysfunction, but often reflects insufficient stress-management strategies and coping resources for dealing with ongoing work demands and emotional strain. From this perspective, a growth mindset may serve as an important personal resource that helps employees better cope with stressful work situations and alleviate workplace anxiety, thereby shifting attention from pathology-oriented explanations toward a resource- and coping-based understanding of anxiety. Anxiety consumes substantial executive control resources, such as attentional regulation and self-regulatory capacity, thereby limiting the cognitive resources available for proactive planning and exploration. When workplace anxiety is alleviated, employees are more likely to conserve cognitive resources for self-initiated, future-oriented action (Bakker et al., 2023), thereby exhibiting higher levels of proactive behavior.

**Figure 1:**
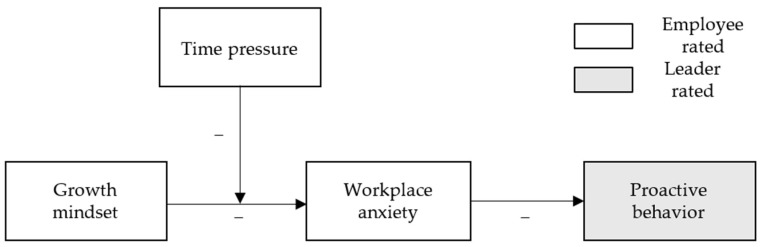
Research model.

Moreover, the extent to which a growth mindset alleviates workplace anxiety, and in turn, promotes proactive behavior, is likely to depend on the work context. According to the JD–R model’s coping hypothesis, challenging job demands can create opportunities for learning and growth, motivating employees to proactively mobilize personal resources and amplifying the beneficial effects of resources on behavior (Bakker et al., 2023; Demerouti et al., 2001). Time pressure—a pervasive challenge demand in contemporary work settings (Prem et al., 2017)—refers to employees’ subjective experience of time scarcity resulting from task deadlines and completion requirements (Maruping et al., 2015). Time pressure can sharpen attentional focus, encourage work process streamlining, and stimulate rapid learning and adaptation. Under conditions of high time pressure, employees are more likely to draw upon a growth mindset as a personal resource to cope with challenges, thereby more effectively alleviating workplace anxiety. Accordingly, time pressure may strengthen the anxiety-buffering effect of growth mindset. Despite its theoretical relevance, this moderating role has not yet been empirically examined. Taken together, based on the JD–R model, this study develops a theoretical model linking employees’ growth mindset to proactive behavior (see Figure 1), and examines the mediating role of workplace anxiety and the moderating role of time pressure in this process.

This study is expected to make three primary theoretical contributions. First, by conceptualizing growth mindset as a capability malleability-based personal resource and linking it to proactive behavior, this study extends the antecedent domain of proactive behavior beyond capability level-based self-evaluations that dominate existing research, thereby offering a more comprehensive account of its underlying resource-based antecedents. In doing so, it introduces a more fundamental class of personal resources grounded in individuals’ implicit beliefs about the malleability of human capability, rather than their current perceived ability to control and influence the environment. Moreover, by examining its direct effect, this study moves beyond the prevailing tendency to treat growth mindset primarily as a moderator and provides evidence of its main effects on employee behavior. Second, grounded in the JD–R model, this study advances a “personal resources–emotional depletion–employee behavior” framework. By identifying workplace anxiety as a mediating mechanism, this research moves beyond broad indicators of psychological resource depletion, such as burnout and emotional exhaustion, and provides a more fine-grained emotional explanation of how a growth mindset translates into proactive behavior. Finally, by introducing time pressure as a contextual factor from the perspective of challenge job demands, this study clarifies the boundary conditions under which a growth mindset influences proactive behavior and offers a direct test of the JD–R model’s coping hypothesis concerning the synergistic effects of job demands and personal resources.

## 2. Theory and Hypotheses

### 2.1. Job Demands–Resources Model

Job demands–resources (JD–R) model conceptualizes job demands as aspects of work that require sustained physical, psychological, or social effort and are therefore associated with certain costs, such as high workload, time pressure, role conflict, and emotional labor (Demerouti et al., 2001). In contrast, job resources refer to physical, psychological, social, or organizational factors that help individuals achieve work goals, promote learning and growth, and reduce the costs associated with job demands, including autonomy, social support, developmental opportunities, and a climate of fairness (Demerouti et al., 2001). Recent extensions of the JD–R model incorporate personal resources into this framework, defining them as individuals’ subjective perceptions of their ability to control and influence their environment (J. Y. Lee et al., 2020). Beyond their association with job resources, personal resources are theorized to shape how individuals interpret and respond to job demands, thereby influencing their psychological functioning and behavioral outcomes. Employees with abundant personal resources tend to adopt more positive perspectives on their work environment and are better able to cope with job demands (Bakker & Demerouti, 2017; Demerouti & Bakker, 2023; Lyddy et al., 2025). As such, personal resources can directly influence individual- and organization-level outcomes through cognitive and emotional processes (Xanthopoulou et al., 2009). Given that growth mindset serves a self-regulatory function, we conceptualize it as an important personal resource, and drawing on the JD–R model, examine its influence on employees’ proactive behavior.

### 2.2. Growth Mindset and Workplace Anxiety

Growth mindset originates from Dweck’s implicit theory framework (Dweck, 1999), which distinguishes between an entity theory—the belief that personal attributes are fixed—and an incremental theory, which views attributes as malleable and capable of development through effort and learning (Rattan & Ozgumus, 2019). In organizational contexts, employee growth mindset is defined as employees’ belief—formed through daily observation and accumulated experience—that their work-related abilities are malleable and can be developed over time (Rattan & Ozgumus, 2019). Prior research suggests that a growth mindset enhances self-regulatory capacity across emotional, cognitive, and behavioral domains by shaping beliefs about effort, attributional responses to setbacks, and goal orientation toward competence development (Keating & Heslin, 2015). Empirically, employee growth mindset has been linked to higher job performance (Ma & Zhu, 2023) and lower levels of counterproductive work behavior (Li et al., 2021). Building on this evidence, we propose that growth mindset may also play a critical role in alleviating employees’ workplace anxiety.

Anxiety is a negative emotional state characterized by tension, worry, and apprehension in response to anticipated future threats and uncertainty (Marteau & Bekker, 1992). Within the JD–R framework, the anxiety emotion represents a strain-based and threat-related response that arises when individuals perceive themselves as unable to effectively cope with job demands. Specifically, when personal resources are inadequate, the health impairment process is activated, leading to heightened strain, whereas abundant resources buffer such impairment (Demerouti et al., 2001). We argue that a growth mindset, as a valuable personal resource (Caniëls et al., 2018), reduces workplace anxiety by shaping how employees interpret and respond to such demands. Specifically, employees with a strong growth mindset are more likely to proactively seek learning opportunities, such as participating in training programs or consulting colleagues, which enhances their sense of competence and reduces uncertainty about future performance (Jeong et al., 2023; Solberg et al., 2024). Moreover, employees high in growth mindset tend to perceive feedback as an opportunity for learning rather than a threat to self-concept (Zingoni & Byron, 2017), prompting more frequent feedback seeking from supervisors and coworkers and fostering a more adaptive interpretation of evaluative situations. In addition, such employees are more likely to express preferences for greater control over their work, thereby strengthening their perceived sense of agency. Finally, employees with a growth mindset are more inclined to view organizational training as an opportunity for development, which reinforces their expectations of improvement and future growth (Tian et al., 2023). According to the JD–R model, psychological strain arises when individuals perceive job demands as exceeding their coping capacity, whereas personal resources help mitigate such burnout-related experiences, such as anxiety (Bakker & Demerouti, 2017). Prior research has similarly demonstrated that greater resources are associated with lower levels of workplace anxiety (Lacerenza et al., 2024). Taken together, we propose that a growth mindset reduces employees’ workplace anxiety by shaping their future-oriented interpretation of job demands and attenuating anticipatory perceptions of threat and uncertainty.

**Hypothesis** **1.** *Employees’ growth mindset negatively predicts subsequent workplace anxiety*.

### 2.3. Mediating Role of Workplace Anxiety

The JD–R model posits that psychological strain resulting from insufficient personal resources undermines employee functioning, whereas strain reduction facilitates positive behavioral outcomes (Demerouti et al., 2001). Building on this logic, we argue that the anxiety-reducing effect of a growth mindset further promotes employees’ proactive behavior. Proactive behavior refers to self-initiated and future-oriented actions through which individuals intentionally seek to change themselves or their environments. It is characterized by three core features: self-initiation, whereby individuals act volitionally rather than in response to external demands; future orientation, which involves attention to long-term issues, anticipation of potential opportunities or challenges, and proactive planning; and change orientation, which entails challenging the status quo and persistently engaging in problem solving to bring about improvement (Bindl & Parker, 2011; Frese & Fay, 2001; Grant & Ashford, 2008).

Research indicates that engaging in proactive behavior requires substantial investments of cognitive resources (e.g., attention and decision-making capacity), emotional resources (e.g., emotion regulation), and physical resources (e.g., energy expenditure) (Salanova & Schaufeli, 2008). Workplace anxiety, however, represents a state of emotional depletion characterized by persistent tension and worry that consumes these limited resources (McCarthy et al., 2016). When anxiety is elevated, their cognitive resources—such as attentional focus and self-regulatory capacity—are continuously taxed (Cheng & McCarthy, 2018), leading them to conserve resources, avoid risk, and refrain from initiating new tasks, thereby inhibiting proactive behavior. In contrast, by reducing workplace anxiety, a growth mindset helps employees maintain cognitive and emotional resources that are essential for self-initiated and future-oriented action. Lower anxiety enhances individuals’ capacity for sustained attention, cognitive flexibility, and behavioral engagement, thereby facilitating proactive behaviors such as proposing improvements, acquiring new skills, and undertaking challenging tasks. Thus, we propose:

**Hypothesis** **2.** *Workplace anxiety mediates the relationship between employees’ growth mindset and proactive behavior*.

### 2.4. Moderating Role of Time Pressure

The JD–R model further suggests that challenge job demands can activate and amplify the positive effects of personal resources (Bakker et al., 2023; Demerouti et al., 2001). Challenge job demands are defined as demands that require substantial effort and skill investment while simultaneously promoting learning and growth (Bakker et al., 2023; Demerouti et al., 2001). Although such demands may elicit short-term strain, they are generally associated with favorable psychological and developmental outcomes. When employees encounter challenge demands, the processes through which they identify, evaluate, and mobilize personal resources are activated, thereby enabling these resources to more effectively alleviate related strain, such as anxiety (Bakker & Demerouti, 2017). In contemporary competitive work environments, efforts to enhance operational efficiency have led to pervasive time pressure, defined as employees’ subjective experience of time scarcity arising from insufficient time to complete work-related tasks. Early research primarily emphasized the detrimental effects of time pressure on employee health and well-being (Roxburgh, 2004). More recent studies, however, conceptualize time pressure as a challenge demand and demonstrate its positive effects on work engagement (Baethge et al., 2018), well-being (Stiglbauer, 2017), and learning behavior (Prem et al., 2017). Therefore, we argue that time pressure, as a challenge job demand, may strengthen the negative relationship between growth mindset and workplace anxiety.

Specifically, under conditions of high time pressure, although tight deadlines may deplete employees’ cognitive resource reserves (Urbach & Weigelt, 2019), they simultaneously induce heightened attentional focus, workflow optimization, enhanced self-management, and rapid learning and adaptation. To sustain task performance in such contexts, employees are more strongly motivated to draw upon and activate internal psychological resources. This heightened motivational state encourages employees to more actively mobilize their growth mindset in coping with work-related challenges. Through more frequent and intensive activation of a growth mindset, employees are better able to reframe job demands as manageable and development-oriented, thereby reducing perceived uncertainty and alleviating workplace anxiety. Conversely, under low time pressure, employees are less likely to perceive their work environment as challenging, resulting in weaker alignment between personal resources and job demands. In such contexts, resource activation and transformation processes are less fully engaged, and employees have reduced motivation to mobilize a growth mindset, thereby rendering its anxiety-buffering effect relatively limited. Therefore, we propose:

**Hypothesis** **3.** *Time pressure strengthens the negative relationship between employees’ growth mindset and workplace anxiety, such that this relationship is stronger under high rather than low time pressure*.

### 2.5. Moderated Mediation Effect

Drawing on the JD–R model, Hypothesis 2 proposes that a growth mindset indirectly influences proactive behavior through workplace anxiety. Specifically, a growth mindset helps employees reframe job demands in a more controllable and development-oriented way, thereby reducing workplace anxiety. Lower levels of anxiety, in turn, preserve individuals’ cognitive and emotional capacity for self-initiated, future-oriented action, which facilitates proactive behavior. Hypothesis 3 further suggests that the first stage of this mediating process is contingent on time pressure, such that the anxiety-reducing effect of a growth mindset varies as a function of time pressure. Integrating these arguments, we propose a moderated mediation model in which time pressure conditions the overall indirect effect of a growth mindset on proactive behavior via workplace anxiety. Specifically, when time pressure is high, a growth mindset is more effective in alleviating workplace anxiety, thereby strengthening its indirect effect on proactive behavior; when time pressure is low, this indirect effect is comparatively weaker. Accordingly, we propose:

**Hypothesis** **4.** *Time pressure strengthens the indirect effect of employees’ growth mindset on proactive behavior via workplace anxiety, such that this indirect effect is stronger under high rather than low time pressure*.

## 3. Methods

### 3.1. Samples and Procedure

Participants were full-time employees working in the Internet and information technology (IT) industries in China. To enhance sample diversity and external validity, we collaborated with Wenjuanxing, a widely used online survey platform in China, to distribute the survey. Accordingly, a convenience sampling approach was adopted, with participation recruited from employees registered on the Wenjuanxing platform who were willing to take part in the study. To ensure data quality, participation was restricted to users with high platform credibility scores, and the platform automatically screened out responses that failed built-in quality checks. Participants receive monetary compensation upon completing each survey, which helped enhance engagement and ensure the accuracy and reliability of the online data collection process. Because participation was voluntary and recruitment relied on an online platform and network-based distribution, the sample was not randomly selected, and potential sample selection bias should be acknowledged.

We conducted a three-wave, multisource lagged research design in this study. To mitigate potential common method bias, several procedural remedies were implemented. First, all surveys were completed anonymously. Second, participants were explicitly informed that there were no right or wrong answers. Third, the order of questionnaire items was counterbalanced to minimize potential order effects. Specifically, items measuring different constructs (e.g., growth mindset, workplace anxiety, proactive behavior, and time pressure) were presented in varying sequences across participants, and the order of items within each construct was randomized. This approach ensured that each item appeared in different positions across respondents, minimizing potential order effects and reducing the likelihood of systematic response patterns (Huang et al., 2026). In addition, data were collected at three time points separated by two-week intervals using a matched employee–supervisor design. At Time 1 (T1; 19 November 2024), employees provided demographic information and completed self-report measures of growth mindset, time pressure, and proactive personality, yielding 418 responses. At Time 2 (T2; 4 December 2024), employees reported their positive and negative affect as well as workplace anxiety, resulting in 364 responses. At Time 3 (T3; 19 December 2024), employees’ proactive behavior was rated by their direct supervisors, yielding 337 responses.

After matching responses across the three waves, 336 employee–supervisor dyads were initially obtained. Following data screening, questionnaires with excessively short completion times or invariant response patterns were excluded. The final sample consisted of 326 valid matched dyads, corresponding to an effective response rate of 77.99%. In the final sample, 66.3% of participants were male, 93.9% held a bachelor’s degree, the average age was 32.12 years (*SD* = 4.41), and the average organizational tenure was 8.23 years (*SD* = 4.33).

### 3.2. Measures

All variables were rated on a five-point Likert scale (1 = strongly disagree, 5 = strongly agree). Same-point Likert scales with different scaling were used for positive and negative affect. The original English scales were translated into Chinese following a translation and back-translation procedure to ensure cross-cultural equivalence. The full list of items is presented in Appendix A.

Growth mindset. Growth mindset was measured using a six-item scale developed by Berg et al. (2023) and was self-reported by employees. Sample items include “I can change my core abilities” and “No matter what kind of person someone is, they can always change a great deal”. The scale demonstrated acceptable construct validity (χ^2^/df = 2.437, CFI = 0.992, TLI = 0.976, RMSEA = 0.066, SRMR = 0.018), and the Cronbach’s α for this scale was 0.779.

Time pressure. Time pressure was assessed with a four-item scale developed by Maruping et al. (2015) and was self-reported by employees. Sample items include “I have often been under a lot of pressure to complete my tasks on time” and “I have not been afforded much time to complete my tasks”. The scale demonstrated acceptable construct validity (χ^2^/df = 1.125, CFI = 0.999, TLI = 0.998, RMSEA = 0.020, SRMR = 0.014), and the Cronbach’s α for this scale was 0.765.

Workplace anxiety. Workplace anxiety was measured using an eight-item scale developed by McCarthy et al. (2016), which was originally adapted from McCarthy and Goffin (2004). Employees self-reported their levels of workplace anxiety. Sample items include “I worry that I will not be able to successfully manage the demands of my job” and “I feel nervous and apprehensive about not being able to meet performance targets”. The scale demonstrated acceptable construct validity (χ^2^/df = 2.343, CFI = 0.998, TLI = 0.989, RMSEA = 0.064, SRMR = 0.007), and the Cronbach’s α for this scale was 0.855.

Proactive behavior. Proactive behavior was measured using a seven-item scale developed by Frese et al. (1997) and was rated by employees’ direct supervisors. Sample items include “Whenever a problem arises, this subordinate immediately seeks a solution” and “Whenever there is an opportunity to participate actively, this subordinate seizes it”. The scale demonstrated acceptable construct validity (χ^2^/df = 1.693, CFI = 0.998, TLI = 0.991, RMSEA = 0.046, SRMR = 0.010), and the Cronbach’s α for this scale was 0.832.

Control variables. We controlled for several demographic variables that may influence proactive behavior, including gender, education, age, and tenure. In addition, because employees’ proactive personality may affect their engagement in proactive behavior (McCormick et al., 2019), we included proactive personality as a control variable. Proactive personality was measured using a ten-item scale developed by Yan et al. (2025). Sample items include “I strongly desire to bring about positive changes for myself” and “I like to think ahead about what problems need to be addressed in advance”. The scale demonstrated overall acceptable construct validity (χ^2^/df = 4.023, CFI = 0.978, TLI = 0.946, RMSEA = 0.096, SRMR = 0.029), and the Cronbach’s α for this scale was 0.827. Given that workplace anxiety served as the mediator in this study, we also controlled for employees’ positive and negative affect. These variables were measured using an eight-item scale developed by Bindl et al. (2012), with four items assessing positive affect and four items assessing negative affect. Sample items include “How often have you felt enthusiastic in your recent daily work?” and “How often have you felt depressed in your recent daily work?”. Responses were rated on a five-point scale ranging from 1 (never) to 5 (always). The positive affect scale demonstrated overall acceptable construct validity (χ^2^/df = 4.756, CFI = 0.975, TLI = 0.924, RMSEA = 0.107, SRMR = 0.027), and the Cronbach’s α was 0.753. The negative affect scale demonstrated acceptable construct validity (χ^2^/df = 1.437, CFI = 0.999, TLI = 0.994, RMSEA = 0.037, SRMR = 0.009), and the Cronbach’s α was 0.802. Although the RMSEA values for proactive personality and positive affect slightly exceeded the conventional threshold of 0.08, the scales were deemed acceptable for use in this study given the known sensitivity of RMSEA to model complexity and sample size, as well as the acceptable CFI and TLI values.

### 3.3. Analytical Strategy

We conducted statistical analyses and hypothesis testing using SPSS 26.0 and Mplus 8.3. First, we assessed the potential threat of common method bias by combining diagnostic procedures implemented in SPSS and Mplus. Second, we performed confirmatory factor analysis (CFA) in Mplus 8.3 to examine the discriminant validity of the focal constructs. Third, we computed descriptive statistics and correlations for all study variables. Finally, we tested the hypothesized model using Mplus 8.3. To examine the moderated mediation effects, we employed a bias-corrected bootstrap procedure with 5000 resamples, and reported unstandardized regression coefficients.

## 4. Results

### 4.1. Confirmatory Factor Analysis

We conducted a confirmatory factor analysis in Mplus 8.3 to assess the discriminant validity of the four focal variables. To improve model fit and reduce the complexity of the measurement model, the eight items measuring anxiety were aggregated into four parcels using a balanced parceling approach (Landis et al., 2000). The results (see Table 1) indicate that the proposed four-factor model provided a significantly better fit to the data than alternative one-factor, two-factor, and three-factor models, demonstrating acceptable fit indices (χ^2^/df = 2.068, CFI = 0.925, TLI = 0.914, RMSEA = 0.057, SRMR = 0.046) that all met the commonly accepted thresholds. These findings provide strong support for the discriminant validity of the constructs.

### 4.2. Common Method Variance

To assess potential common method bias (Podsakoff et al., 2003), we adopted multiple diagnostic procedures. First, Harman’s single-factor test was conducted using exploratory factor analysis in SPSS. The results indicate that the first unrotated factor accounted for 31.21% of the total variance, below the commonly accepted threshold of 40%, suggesting that common method bias was unlikely to be a major concern. Second, a single-factor confirmatory factor analysis was performed in which all measurement items of the focal constructs were loaded onto a single latent factor. The model showed poor fit (χ^2^/df = 5.435, CFI = 0.680, TLI = 0.645, RMSEA = 0.117, SRMR = 0.084), indicating that the observed relationships could not be attributed to a single common method factor. Finally, we applied the unmeasured latent method construct (ULMC) approach (see Table 1) by estimating two competing models: one without a latent method factor (χ^2^/df = 2.068, CFI = 0.925, TLI = 0.914, RMSEA = 0.057, SRMR = 0.046), and one with an additional latent method factor (χ^2^/df = 2.919, CFI = 0.866, TLI = 0.846, RMSEA = 0.077, SRMR = 0.162). The inclusion of the latent method factor worsened model fit, indicating that common method variance did not substantially account for the observed relationships. Collectively, these results suggest that common method bias is unlikely to threaten the validity of the findings.

### 4.3. Descriptive Statistics and Correlations

Table 2 presents the means, standard deviations, and correlations among the variables. As shown in Table 2, growth mindset was significantly and negatively correlated with workplace anxiety (*r* = −0.438, *p* < 0.001), and workplace anxiety was significantly and negatively correlated with proactive behavior (*r* = −0.519, *p* < 0.001). In contrast, growth mindset was significantly and positively correlated with proactive behavior (*r* = 0.465, *p* < 0.001). In addition, time pressure was positively associated with workplace anxiety (*r* = 0.486, *p* < 0.001). Overall, these results provide preliminary support for the proposed hypotheses.

### 4.4. Hypothesis Testing

Test of main effect. Hypothesis 1 proposed that employees’ growth mindset negatively predicts workplace anxiety. To test this, we conducted regression analyses controlling for gender, education, age, tenure, proactive personality, positive affect, and negative affect. As shown in Table 3, growth mindset was significantly and negatively related to workplace anxiety (*B* = −0.189, *SE* = 0.058, *p* = 0.001), thus supporting Hypothesis 1.

Test of mediation effect. Table 3 also showed that workplace anxiety was, in turn, negatively associated with proactive behavior (*B* = −0.242, *SE* = 0.070, *p* = 0.001), providing preliminary support for the proposed mediating role of workplace anxiety. We further conducted the mediation test using bias-corrected bootstrap analyses with 5000 resamples to estimate the indirect effect. The results indicate that workplace anxiety significantly mediated the relationship between growth mindset and proactive behavior (indirect effect = 0.046, *SE* = 0.019, *p* = 0.017). The 95% confidence interval for the indirect effect was [0.016, 0.096], which did not include zero. Taken together, these results provide robust support for Hypothesis 2, suggesting that growth mindset enhances proactive behavior by alleviating employees’ workplace anxiety.

**Figure 2:**
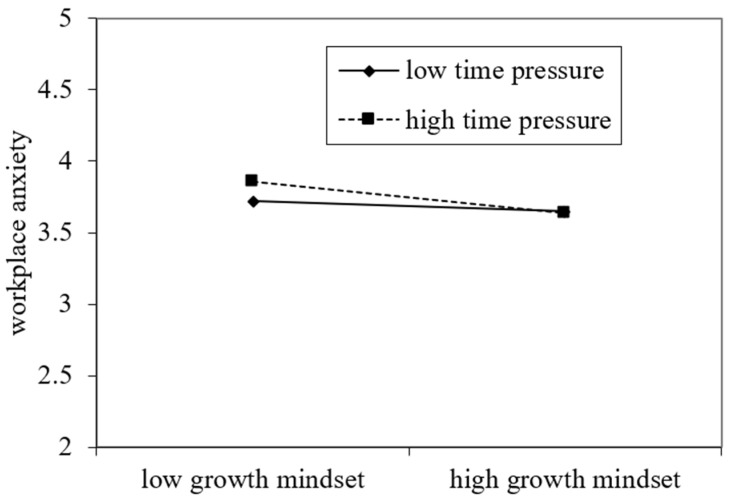
Moderating effect of time pressure on the relationship between employee growth mindset and workplace anxiety (simple slopes analysis).

Test of moderation effect. Hypothesis 3 proposed that time pressure strengthens the negative relationship between employees’ growth mindset and workplace anxiety. Prior to testing the moderation effect, growth mindset and time pressure were grand-mean centered to reduce potential multicollinearity (Dalal & Zickar, 2012). As reported in Table 4, the regression results revealed a significant negative interaction between growth mindset and time pressure in predicting workplace anxiety (*B* = −0.122, *SE* = 0.045, *p* = 0.007), providing support for Hypothesis 3. To further interpret this interaction, we conducted simple slope analyses at high (+1 SD) and low (−1 SD) levels of time pressure (see Figure 2). The results indicate that when time pressure was high, growth mindset was significantly associated with lower workplace anxiety (*B* = −0.214, *SE* = 0.061, *t* = −3.504, *p* < 0.001). In contrast, when time pressure was low, the relationship between growth mindset and workplace anxiety was nonsignificant (*B* = −0.072, *SE* = 0.067, *t* = −1.078, *p* = 0.281).

**Figure 3:**
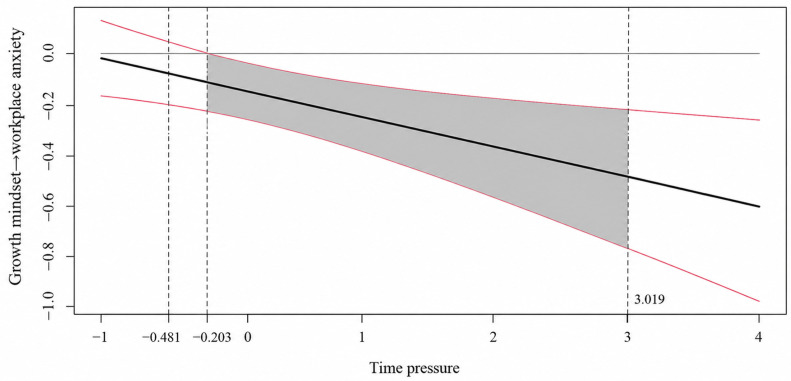
Moderating effect of time pressure on the relationship between employee growth mindset and workplace anxiety (Johnson–Neyman method). Note. The black solid line represents the estimated conditional effect of growth mindset on workplace anxiety across levels of time pressure. The upper and lower red lines indicate the upper and lower bounds of the 95% confidence interval, respectively.

To overcome the limitations of the pick-a-point approach, we further applied the Johnson–Neyman technique to identify the region of significance for the conditional effect of growth mindset across levels of time pressure (see Figure 3). The Johnson–Neyman results showed that within the observed range of time pressure in the sample (mean-centered; −0.481 to 3.019), the conditional effect of growth mindset on workplace anxiety became statistically significant when time pressure exceeded −0.203. Below this threshold, the effect was nonsignificant, whereas above it, the confidence intervals lay entirely below zero. Together, these findings provide robust evidence that time pressure strengthens the anxiety-reducing effect of growth mindset.

Test of moderated mediation effect. Hypothesis 4 predicted a moderated mediation effect, such that time pressure would amplify the indirect effect of growth mindset on proactive behavior via workplace anxiety. This hypothesis was tested using bias-corrected bootstrap analyses with 5000 resamples (see Table 5). When time pressure was high (+1 SD), the indirect effect of growth mindset on proactive behavior through workplace anxiety was significant (indirect effect = 0.037, *SE* = 0.018, 95% CI = [0.009, 0.084]). In contrast, when time pressure was low (−1 SD), the indirect effect was not significant (indirect effect = 0.012, *SE* = 0.012, 95% CI = [−0.005, 0.051]). Moreover, the difference between the indirect effects at high versus low levels of time pressure was statistically significant (Δ indirect effect = 0.024, *SE* = 0.013, 95% CI = [0.007, 0.055]), with a 95% confidence interval that did not include zero. Collectively, these results indicate that time pressure strengthens the mediating role of workplace anxiety in the relationship between growth mindset and proactive behavior, thereby supporting Hypothesis 4.

Overall, the hypothesis testing results are summarized in Table 6. As shown, all hypotheses were empirically supported, with growth mindset significantly predicting workplace anxiety (H1), workplace anxiety mediating the relationship between growth mindset and proactive behavior (H2), time pressure moderating the relationship between growth mindset and workplace anxiety (H3), and a significant moderated mediation effect of time pressure on the indirect effect of growth mindset via workplace anxiety (H4).

## 5. Discussion

Drawing on the JD–R model, this study developed and tested a moderated mediation model to clarify the mechanisms and boundary conditions through which employees’ growth mindset influences proactive behavior. Using three-wave matched employee–supervisor data from 326 dyads, we found that employees’ growth mindset was negatively associated with workplace anxiety, which in turn promoted proactive behavior. Moreover, time pressure amplified the anxiety-buffering effect of growth mindset and strengthened its indirect effect on proactive behavior via workplace anxiety. Specifically, when time pressure was high, the indirect effect of growth mindset on proactive behavior through reduced workplace anxiety was more pronounced. By conceptualizing growth mindset as a capability malleability-based personal resource within the JD–R framework, this study advances understanding of the antecedents of proactive behavior and provides practical insights for managers seeking to foster employee proactivity.

### 5.1. Theoretical Implications

This study makes three theoretical contributions. First, it advances our understanding of the antecedents of proactive behavior by foregrounding growth mindset as a capability malleability-based personal resource grounded in implicit beliefs about the malleability of human capability, rather than in the current perceived ability to control and influence the environment. Prior research has predominantly emphasized external work resources—such as leadership styles and organizational systems—in explaining employee proactivity, including transformational leadership (Schmitt et al., 2016), empowering leadership (Schilpzand et al., 2018), inclusive leadership (Xu et al., 2021), and high-performance work systems (Lin et al., 2022). In contrast, personal resources have received comparatively less attention. Existing studies on personal resources have predominantly focused on capability level-based resources that reflect individuals’ current perceived ability to cope with and regulate their work environments, such as resilience (Caniëls & Baaten, 2019), awareness regulation capacity (Hu et al., 2025), and self-efficacy (Abuelhassan & AlGassim, 2022), whereas relatively less attention has been paid to capability malleability-based personal resources that shape individuals’ fundamental assumptions about human capability—a growth mindset. By conceptualizing growth mindset as a personal resource that facilitates proactive behavior, this study addresses this gap and provides a more comprehensive resource-based perspective on the antecedents of proactive behavior. Beyond identifying growth mindset as an antecedent, this study further extends the growth mindset literature by examining its direct effects. While previous research has primarily treated growth mindset as a moderating variable (Cunningham et al., 2023), its main effects in organizational settings remain insufficiently examined. Responding to Rattan and Ozgumus’s (2019) call for greater attention to these effects, this study demonstrates that a growth mindset directly predicts proactive behavior. In doing so, it broadens the range of outcomes associated with a growth mindset beyond previously examined consequences such as job performance (Ma & Zhu, 2023) and counterproductive work behavior (Li et al., 2021).

Second, this study clarifies the mechanisms linking growth mindset to proactive behavior by drawing on the JD–R model. Previous studies have largely relied on general mindset theory or social cognitive theory to explain how a growth mindset influences employee behavior (Feng et al., 2023; Solberg et al., 2024). For instance, a growth mindset has been found to promote organizational citizenship behavior by enhancing moral control (Feng et al., 2023), and self-efficacy has been also identified as a key mediator (Burnette et al., 2020). However, these studies have largely overlooked the emotional mechanism through which a growth mindset influences behavioral outcomes. In response, this study conceptualizes growth mindset as a personal resource that directly shapes how employees understand and respond to job demands, thereby reducing workplace anxiety and facilitating proactive behavior. This finding not only enriches our understanding of the mechanism through which a growth mindset affects proactive behavior, but also extends the application of the JD–R model. Prior JD–R research has typically treated general burnout or emotional exhaustion as the mediating mechanism linking personal resources to outcomes (Huynh et al., 2014), without examining specific emotional states. By focusing on workplace anxiety as the mediator, this study addresses this gap and provides a more fine-grained emotional account of the psychological mechanism. Moreover, by conceptualizing workplace anxiety as an anticipatory emotional response rooted in perceived coping inadequacy rather than clinical psychopathology, this study offers a resource-based, non-pathologizing perspective on employee anxiety. This framing aligns with recent arguments that subclinical anxiety in work settings often reflects insufficient stress-management resources rather than underlying psychological dysfunction (e.g., McCarthy et al., 2016). Accordingly, our findings suggest that workplace anxiety may be more responsive to resource-based interventions—such as cultivating employees’ growth mindset—than traditional deficit-oriented or therapeutic approaches would assume. This shifts the scholarly discourse from treating workplace anxiety as an individual pathology to understanding it as a malleable, context-dependent strain state that can be mitigated through personal resource development.

Third, this study identifies the boundary conditions under which a growth mindset influences proactive behavior. The JD–R model suggests that challenging job demands can activate the positive effects of personal resources by motivating employees to mobilize these resources more intensively (Demerouti et al., 2001). Although theory implies that certain challenge demands may strengthen the effects of a growth mindset, empirical research has rarely specified which demands play this role. Focusing on time pressure as a prototypical challenge demand, this study shows that the indirect effect of growth mindset on proactive behavior via reduced workplace anxiety is stronger under high time pressure. This finding clarifies the boundary conditions of growth mindset effects and provides direct empirical support for the JD–R model’s coping hypothesis, highlighting the importance of the alignment between personal resources and job demands. These results are also consistent with prior findings. For example, Ma and Zhu (2023) showed that employees with a growth mindset can better activate its positive effects under negative external conditions, such as underdog expectations, and Zhang et al. (2024) demonstrated that the value of personal resources becomes more salient when employees face challenging or constrained work environments.

### 5.2. Practical Implications

This study offers several important practical implications. First, organizations should deliberately cultivate employees’ growth mindset as a key driver of proactive behavior. Our findings highlight the pivotal role of employees’ growth mindset in fostering proactive behavior, offering actionable guidance for contemporary talent management and development. Rather than relying solely on static assessments of current competencies, organizations should shift toward evaluating employees’ developmental potential, with greater emphasis on indicators of growth mindset such as learning orientation, developmental aspirations, challenge seeking, and tolerance for risk. At the organizational level, managers are encouraged to cultivate a growth mindset culture by moving away from fixed-mindset norms that privilege innate ability and toward a development-oriented culture that values learning and continuous improvement. Such a culture can be reinforced through growth-oriented training initiatives (e.g., deliberate practice programs), the design of developmental career pathways, and reward systems that recognize learning and improvement (e.g., awards for “most improved performance”). At the leadership level, supervisors can further strengthen growth-oriented beliefs by replacing trait-based evaluations with process-focused feedback that emphasizes the link between effort, learning, and outcomes. At the individual level, employees may actively develop a growth mindset through metacognitive practices, such as reflective journaling and cognitive reappraisal.

Second, managers should implement targeted interventions to reduce workplace anxiety, thereby facilitating the translation of employees’ growth mindset into proactive behavior. This study identifies workplace anxiety as a key mechanism linking growth mindset to proactive behavior, underscoring the importance of complementary organizational practices aimed at anxiety reduction. In practice, organizations may establish a multi-level anxiety management system. For example, regular emotional check-ins can be used to monitor employees’ psychological states and incorporate mental health indicators into organizational health diagnostics. When anxiety levels exceed predefined thresholds, organizations can provide timely support through targeted micro-learning resources or peer-support mechanisms. In addition, managers may offer emotion regulation workshops that equip employees with effective strategies—such as cognitive reappraisal—to manage negative emotions and enhance their capacity to cope with emotional strain. By alleviating anxiety, such interventions help preserve the psychological resources necessary for proactive behavior.

Finally, managers should pay close attention to the alignment between employees’ growth mindset and time pressure when allocating tasks and designing work. Our findings suggest that the positive effects of a growth mindset on proactive behavior are amplified under conditions of high time pressure. This indicates that in high-intensity work contexts where time pressure is pervasive and difficult to eliminate, fostering employees’ growth mindset may serve as a relatively low-cost and sustainable buffer against anxiety. Importantly, this result should not be interpreted as advocating the deliberate creation of time pressure to enhance proactivity. Rather, it highlights a critical managerial consideration: employees with a high growth mindset, due to their richer psychological resource reserves, are better equipped to withstand the strain associated with demanding work conditions, whereas employees with a low growth mindset may experience greater difficulty adapting due to resource depletion. Accordingly, managers should carefully calibrate task demands and time constraints to ensure an appropriate fit. Employees with a high growth mindset may be assigned more challenging tasks with reasonable deadlines to activate their developmental potential, whereas employees with a lower growth mindset may require additional support and temporal buffers to perform effectively.

### 5.3. Limitations and Future Directions

This paper inevitably has some limitations. First, the research design constrains the strength of causal inferences. Although we employed a three-wave, multi-source data collection strategy to mitigate common method bias and further verified that it was not a serious concern using Harman’s single-factor test and the ULMC approach, the largely non-experimental nature of the design still limits our ability to draw definitive causal conclusions. Notably, prior growth mindset research has increasingly adopted intervention-based designs (Burnette et al., 2020; Rogers et al., 2023), which offer distinct advantages for causal inference by randomly assigning participants to experimental and control conditions and tracking changes in outcomes over time. Accordingly, future studies could adopt experimental or quasi-experimental designs in organizational settings. For example, researchers might develop structured growth mindset interventions (e.g., multi-week training programs) and examine their causal effects on employees’ proactive behavior using longitudinal or field-experimental approaches.

Second, the generalizability of our findings may be constrained by the characteristics of our sample. Participants were recruited exclusively from the Internet and information technology (IT) industries in China using a convenience sampling approach via an online survey platform (Wenjuanxing). The IT sector is characterized by high-paced work environments, frequent role transitions, and strong performance-orientation cultures, which may render employees particularly attuned to time pressure and developmental demands. Consequently, the moderating effect of time pressure observed in this study may be more pronounced in our sample than in other occupational contexts. Furthermore, China’s collectivistic cultural context, with its emphasis on self-improvement and adaptability, may shape how employees interpret and respond to growth mindset interventions differently than in individualistic cultures. Future research should therefore replicate our model in diverse occupational settings (e.g., healthcare, manufacturing, education) and cultural contexts to establish the external validity of our findings. Relatedly, future studies may adopt probability-based sampling strategies (e.g., stratified random sampling) to enhance sample representativeness and reduce selection bias.

Third, the boundary conditions under which a growth mindset exerts its effects merit further investigation. Drawing on the JD–R model, this study focused on time pressure as a challenge job demand and found that higher time pressure amplifies the beneficial effects of a growth mindset. Building on this logic, other types of challenge job demands may produce similar moderating effects. For example, job complexity represents a salient challenge demand that requires continuous learning and adaptive effort. Prior research suggests that agile work practices are more effective in fostering team work engagement under conditions of high job complexity (Junker et al., 2025). By extension, it is plausible that a growth mindset, as a personal resource, becomes particularly influential when employees face high levels of job complexity. Future research could empirically test this possibility. In addition to job demands, future research should also consider the moderating role of job resources. Existing research indicates that personal resources and job resources jointly shape employee outcomes (Caniëls et al., 2018). From this perspective, organizational-level resources deserve greater scholarly attention. Specifically, factors such as an organizational learning culture, performance evaluation systems (e.g., process-based versus outcome-based appraisal), and developmental opportunities provided by the organization (e.g., job rotation programs and mentoring systems) may constitute critical contextual resources that enable employees to more effectively translate a growth mindset into tangible work-related gains (Rattan & Ozgumus, 2019). Investigating how these organizational resources interact with a growth mindset would further deepen our understanding of when a growth mindset promotes proactive behavior.

## Figures and Tables

**Table 1:** Results of confirmatory factor analysis.

Models	χ^2^/df	CFI	TLI	RMSEA	SRMR
Five-factor model (A, B, C, D, E)	2.919	0.866	0.846	0.077	0.162
Four-factor model (A, B, C, D)	2.068	0.925	0.914	0.057	0.046
Three-factor model (A + B, C, D)	3.158	0.847	0.827	0.081	0.061
Two-factor model (A + B + C, D)	4.420	0.755	0.726	0.102	0.078
One-factor model (A + B + C + D)	5.435	0.680	0.645	0.117	0.084

Note. A = growth mindset; B = time pressure; C = workplace anxiety; D = proactive behavior; E = common method factor.

**Table 2:** Means, standard deviations, and correlations.

Variables	1	2	3	4	5	6	7	8	9	10	11
1. Gender											
2. Age	0.040										
3. Education	0.069	0.150 **									
4. Tenure	0.005	0.957 ***	0.022								
5. Proactive personality	0.024	0.096	0.062	0.078							
6. Positive affect	0.034	0.072	0.002	0.071	0.196 ***						
7. Negative affect	−0.027	−0.062	−0.003	−0.067	−0.300 ***	−0.552 ***					
8. Growth mindset	−0.003	0.127 *	0.048	0.121 *	0.753 ***	0.301 ***	−0.382 ***				
9. Workplace anxiety	0.045	−0.052	−0.017	−0.047	−0.328 ***	−0.684 ***	0.546 ***	−0.438 ***			
10. Proactive behavior	−0.025	0.052	0.022	0.077	0.397 ***	0.436 ***	−0.511 ***	0.465 ***	−0.519 ***		
11. Time pressure	0.024	−0.126 *	0.042	−0.135 *	−0.274 ***	−0.485 ***	0.466 ***	−0.410 ***	0.486 ***	−0.520 ***	
M	1.340	32.120	2.030	8.230	3.934	4.091	1.765	3.916	1.693	3.917	1.731
SD	0.474	4.410	0.246	4.330	0.494	0.615	0.630	0.520	0.509	0.555	0.580

Note: *N* = 326; * *p* < 0.05, ** *p* < 0.01, *** *p* < 0.001. gender: 1 = male, 2 = female. education: 1 = associate degree or below, 2 = bachelor’s degree, 3 = master’s degree, and 4 = doctoral degree or above.

**Table 3:** Results of mediating effect.

	Workplace Anxiety (Mediating Variable)			Proactive Behavior (Outcome Variable)		
	*b*	*SE*	*p*	*b*	*SE*	*p*
Control variables						
Gender	0.075	0.040	0.063	−0.020	0.051	0.692
Age	−0.007	0.016	0.667	−0.049	0.021	0.017
Education	−0.004	0.085	0.966	0.120	0.107	0.263
Tenure	0.010	0.017	0.539	0.050	0.021	0.017
Proactive personality	−0.030	0.058	0.606	0.131	0.073	0.075
Positive affect	−0.437	0.037	0.000	0.069	0.056	0.212
Negative affect	0.142	0.037	0.000	−0.220	0.048	0.000
Independent variable						
Growth mindset	−0.189	0.058	0.001	0.174	0.073	0.018
Mediating variable						
Workplace anxiety				−0.242	0.070	0.001
Intercept	4.137	0.427	0.000	4.189	0.608	0.000
*R* ^2^	0.115	0.009	0.000	0.182	0.014	0.000

**Table 4:** Results of moderating effect.

	Workplace Anxiety (Mediating Variable)			Proactive Behavior (Outcome Variable)		
	*b*	*SE*	*p*	*b*	*SE*	*p*
Control variables						
Gender	0.065	0.040	0.101	−0.006	0.048	0.897
Age	−0.009	0.016	0.564	−0.046	0.020	0.019
Education	−0.004	0.084	0.961	0.137	0.102	0.181
Tenure	0.012	0.016	0.454	0.046	0.020	0.022
Proactive personality	−0.016	0.058	0.788	0.121	0.070	0.086
Positive affect	−0.380	0.040	0.000	−0.013	0.055	0.820
Negative affect	0.093	0.039	0.018	−0.138	0.048	0.004
Independent variable						
Growth mindset	−0.143	0.058	0.014	0.085	0.072	0.235
Moderating variable						
Time pressure	0.055	0.041	0.187	−0.197	0.050	0.000
Interaction						
Growth mindset × Time pressure	−0.122	0.045	0.007	0.170	0.056	0.002
Mediating variable						
Workplace anxiety				−0.172	0.067	0.011
Intercept	3.251	0.450	0.000	4.883	0.590	0.000
*R* ^2^	0.111	0.009	0.000	0.165	0.013	0.000

**Table 5:** Moderated mediation effect.

Time Pressure Condition	Indirect Effect (*SE*)	95% CI
High	0.037 (0.018)	[0.009, 0.084]
Low	0.012 (0.012)	[−0.005, 0.051]
Difference	0.024 (0.013)	[0.007, 0.055]

**Table 6:** Summary of hypothesis testing results.

Hypothesis	Relationship	Analysis Type	Results	Supported
H1	Growth mindset → Workplace anxiety	Regression analysis	*B* = −0.189, *SE* = 0.058, *p* = 0.001	Yes
H2	Growth mindset → Workplace anxiety → Proactive behavior	Bias-corrected bootstrap mediation (5000 resamples)	Indirect effect = 0.046, *SE* = 0.019, 95% CI = [0.016, 0.096]	Yes
H3	Growth mindset × Time pressure → Workplace anxiety	Regression analysis (interaction term analysis)	*B* = −0.122, *SE* = 0.045, *p* = 0.007	Yes
H4	Moderated mediation by time pressure	Bias-corrected bootstrap moderated mediation (5000 resamples)	Δ indirect effect = 0.024, *SE* = 0.013, 95% CI = [0.007, 0.055]	Yes

## Data Availability

Data are available from the first author upon reasonable request due to ethical and privacy restrictions.

## Appendix A. Measurement Items

Growth Mindset (from Berg et al., 2023)

1. I can change my core abilities.
2. People can substantially change the kind of person they are.
3. People can do things differently, and even the important parts of who they are can change.
4. I have control over the development of my talent.
5. No matter what kind of person someone is, they can always change a great deal.
6. People can change even their most basic qualities.

Time pressure (from Maruping et al., 2015)

1. I have often been under a lot of pressure to complete my tasks on time.
2. I have not been afforded much time to complete my tasks.
3. The amount of time provided to complete my tasks has been short.
4. Task durations have often been short.

Workplace anxiety (from McCarthy et al., 2016)

1. I am overwhelmed by thoughts of doing poorly at work.
2. I worry that my work performance will be lower than that of others at work.
3. I feel nervous and apprehensive about not being able to meet performance targets.
4. I worry about not receiving a positive job performance evaluation.
5. I often feel anxious that I will not be able to perform my job duties in the time allotted.
6. I worry about whether others consider me to be a good employee for the job.
7. I worry that I will not be able to successfully manage the demands of my job.
8. Even when I try as hard as I can, I still worry about whether my job performance will be good enough.

Proactive behavior (from Frese et al., 1997)

1. This subordinate actively solves problems.
2. Whenever a problem arises, this subordinate immediately seeks a solution.
3. Whenever there is an opportunity to participate actively, this subordinate seizes it.
4. This subordinate takes initiative immediately, even when others do not act.
5. This subordinate quickly exploits opportunities to achieve my objectives.
6. This subordinate usually does more than is formally required.
7. This subordinate is particularly effective at implementing ideas.

Proactive personality (from Yan et al., 2025)

1. I strongly desire to bring about positive changes for myself.
2. I am full of positive expectations for the future.
3. When I anticipate a positive change, I will do everything I can to promote it.
4. I always think about what good development opportunities the future may hold.
5. I enjoy paying attention to opportunities that are beneficial to me.
6. I pay close attention to and try to avoid things that may have a negative impact on me.
7. To avoid potential future losses, I put in my best effort to solve possible problems.
8. I like to think ahead about what problems need to be addressed in advance.
9. When I notice a problem, I habitually act immediately to resolve it.
10. I am always attentive to potential adverse situations.

Positive and negative affect (Bindl et al., 2012)

Instructions: Please indicate how frequently you experience the following emotions in your recent daily work, on a scale from 1 (Never) to 5 (Always).

Positive affect

1. Enthusiastic.
2. Excited.
3. Inspired.
4. Joyful.

Negative affect

1. Depressed.
2. Dejected.
3. Despondent.
4. Hopeless.
